# A multicenter feasibility study on implementing a brief mindful breathing exercise into regular university courses

**DOI:** 10.1038/s41598-023-34737-0

**Published:** 2023-05-16

**Authors:** Annika C. Konrad, Veronika Engert, Reyk Albrecht, Christian Dobel, Nicola Döring, Jens Haueisen, Olga Klimecki, Mike Sandbothe, Philipp Kanske

**Affiliations:** 1 Clinical Psychology and Behavioral Neuroscience, Institute of Clinical Psychology and Psychotherapy, Technische Universität Dresden, Chemnitzer Str. 46, 01187 Dresden, Germany; 2 Institute of Psychosocial Medicine, Psychotherapy, and Psychooncology, Jena University Hospital, Jena, Germany; 3 Faculty of Social and Behavioral Sciences, Friedrich Schiller University Jena, Jena, Germany; 4 Department of Otorhinolaryngology, Jena University Hospital, Jena, Germany; 5 Institute of Media and Communication Science, Ilmenau University of Technology, Ilmenau, Germany; 6 Institute of Biomedical Engineering and Informatics, Ilmenau University of Technology, Ilmenau, Germany; 7 Department of Social Work, Ernst Abbe University of Applied Sciences Jena, Jena, Germany

**Keywords:** Disease prevention, Public health

## Abstract

Practicing mindfulness is associated with stress reduction and with positive effects in the context of learning and teaching. Although effects on student populations have been studied extensively, there are few studies implementing mindfulness exercises in university courses directly. For this reason, we aimed to investigate whether the use of a brief mindfulness exercise in regular university courses, guided by the lecturers, is feasible and has immediate effects on the students’ mental states. We conducted a preregistered multicenter study with one observational arm, following an ABAB design. In total, *N* = 325 students from 19 different university courses were included at baseline and *n* = 101 students at post measurement. Students were recruited by *N* = 14 lecturers located in six different universities in Germany. Lecturers started their courses either by guiding a brief mindfulness exercise (intervention condition) or as they regularly would, with no such exercise (control condition). In both conditions, the mental states of students and lecturers were assessed. Over the semester, *n* = 1193 weekly observations from students and *n* = 160 observations from lecturers were collected. Intervention effects were analyzed with linear mixed-effects models. The brief mindfulness exercise, compared to no such exercise, was associated with lower stress composite scores, higher presence composite scores, higher motivation for the courses, as well as better mood in students. Effects persisted throughout a respective course session. Lecturers also reported positive effects of instructing mindfulness. Implementing a brief mindfulness exercise in regular university teaching sessions is feasible and has positive effects on both students and lecturers.

## Introduction

Ever since Western psychology has embraced mindfulness from Buddhist traditions as a research subject, the number of studies on mindfulness practices has been increasing rapidly^1, 2^. Within this process, the term mindfulness has shifted away from its Buddhist meaning, that involves the (spiritual) cultivation of present-moment awareness of suffering and achievement of insight and wisdom, to a secular conceptualization^2, 3^. From this secular point of view, mindfulness refers, first, to a mental state (and process) in which a person is or becomes aware of their body, mind, and environment in the present moment, without influencing the perceptual state through judgments, emotions, or memories^2^. Second, the term mindfulness is used to refer to formal and informal secular meditation practices, and third, mindfulness can be seen as a “modern treatment trend” in counselling and therapy aiming to help patients^2^. Alongside the conceptual shift, the structured and group-based forms of implementing mindfulness practices, such as Mindfulness-Based Stress Reduction (MBSR;^4, 5^) and Mindfulness-Based Cognitive Therapy (MBCT;^6, 7^), have been extended by online-based self-help programs^8, 9^. Multiple studies have demonstrated positive effects of different forms of mindfulness-based interventions in various samples (for a systematic review of meta-analyses, see^10^), for instance in people with mental disorders^11–13^, healthy people^5^, employees^14^, elderly^15^, or children and adolescents^16^. Positive effects encompass—among others—the reduction of stress and anxiety, as well as an enhancement of well-being and mindfulness^5, 8, 16^. Regarding cognitive domains, small positive effects on working memory and executive function have been found across studies (for meta-analysis see^17^).

Because the benefits of mindfulness-based interventions are wide-ranging in emotional and cognitive domains, mindfulness has also slowly seeped into the context of universities with the aim of stress reduction^18^. For adolescents and young adults in particular, the transition from high school into college or university involves significant life changes, therefore, high stress levels in students have been highlighted as a major issue within higher education for several years now^19–21^. Academic responsibilities and demands, finances, anxiety, poor life-domain balance, research pressures, or self-imposed stress are just some of the strains regularly experienced by students^22, 23^. Certain vulnerabilities seem to come along with higher stress levels in students; for instance, higher levels of neuroticism were positively associated with depressive symptoms and stress, and negatively associated with well-being^24^ and trait mindfulness^25^. In turn, stress and mental health problems are associated with negative consequences like lower academic performance and functioning, as well as drop-out intentions^26–28^.

By implementing stress reduction interventions, such as mindfulness-based interventions, multiple attempts have been made to improve students’ stress coping, reduce their stress, and prevent, or at least diminish, the negative impacts of their increased stress load (for reviews and meta-analyses see^9, 18, 29–31^). After practicing mindfulness between three and 12 weeks, students showed a reduction in depression symptoms^30^ and stress^18^, as well as higher verbal creativity^32^. Improvements in a word retrieval task could be shown even after a single session of mindful breathing^33^. Considering that studying is a cognitively demanding and—for many students—stressful process, the application of mindfulness interventions in the university context seems auspicious.

Although mindfulness-based interventions are well investigated and effective in university students, common barriers for students to use coping strategies are lack of time and high costs^22^. Facing the Coronavirus disease (COVID-19) pandemic, additional barriers, such as contact restrictions, made the access to conventional group-based in-person mindfulness interventions more difficult. Yet, by posing new stressors for students (e.g., contact restrictions, or transition of lectures to online format^34^), and hampering their attention and motivation when attending online courses^35^, the pandemic even increased the importance of accessible coping strategies.

Two potential solutions to overcome such barriers are brief as well as self-help mindfulness-based interventions that are both cost- and time-efficient, and easily accessible. Meta-analyses investigating such brief mindfulness interventions^36^ and self-help mindfulness interventions^37^, reported positive effects on affect, depression, and anxiety in students^36, 37^.

However, although these studies do offer a way to overcome barriers in reaching students, few studies have implemented brief mindfulness interventions directly within the students’ curricula^29^. Yet, doing so would have several advantages. It would be time- and cost-efficient, and feasible for both online and in-person courses. Also, the implementation of a short mindfulness exercise, for example, a 3-min breathing space as introduced by MBCT (e.g.,^38^), at the beginning of a course could positively affect the quality of students’ learning directly (compared to general positive changes resulting from meditation throughout the week). Last, such an intervention could strengthen the students’ sense of closeness with lecturers and fellow students with whom they share the meditation experience. Overall, while the general effectiveness of brief mindfulness interventions has clearly been shown, several open questions remain: First, does the use of mindfulness interventions in regular university courses show immediate positive effects on students’ well-being and mental states (e.g., concentration, perceived stress), and second, how do students (and lecturers) evaluate such a low-intensity offer?

To address these questions, we implemented a brief (3- to 4-min) mindful breathing exercise for one semester at several German universities. The exercise was guided biweekly by the lecturers at the beginning of regular university courses. By conducting a feasibility and effectiveness study of high ecologic validity, we may help advising universities on how to realize time-efficient, cost-efficient, and easily accessible intervention strategies for students. Informing about students’ and lecturers’ acceptance and evaluation of such an offer may also help reduce potential pitfalls in future implementations.

Our study was preregistered (https://osf.io/nxucw) with the following main hypotheses and different exploratory research questions:

- H1: The application of a short mindfulness exercise at the beginning of a university course, compared to no such exercise, will have an immediate positive effect on students’ mental states (specifically on self-rated concentration, presence, alertness, distraction by thoughts, energy, stress perception, mood).
- H2: Said positive effect will persist throughout the teaching session.
- We asked the following exploratory research questions:
- E1: Do other factors influence the mental state of university students at the beginning of a teaching session (trait mindfulness, sense of stress, trait attentional control, personality traits, and prior experience in meditation)?
- E2: Do lecturers’ well-being and authenticity during their instruction, and the level of standardization of the given mindfulness instruction have an impact on students' mental state after the instruction?
- E3: Does the instruction of a short mindfulness exercise at the beginning of a lecture (compared to lectures without such an exercise) have an effect on the immediate mental state of the instructing lecturers?
- E4: How do university lecturers and students evaluate the short mindfulness exercise in terms of its effectiveness at the end of the semester?
- E5: Do other factors (such as participation, online vs. in-person instruction, or individual evaluation of the exercise) influence the overall evaluation of the mindfulness exercise at the end of the semester?

## Method

### Recruitment

The study complied with the ethical standards of the Declaration of Helsinki^39^and was approved by two German ethics committees (Technische Universität Dresden: SR-EK-270052021; Friedrich-Schiller Universität Jena: 2021-2121-BO).

During the lecture-free period between the winter semester of 2020/2021 and the summer semester of 2021, lecturers willing to participate in the study were recruited via mailing lists and personal contacts of the Mindful Universities network. Inclusion criteria for lecturers were the participation in an online workshop led by a certified MBSR senior teacher (up to three appointments of 2 h each), the willingness to implement mindfulness exercises and a control condition in at least two teaching sessions each. Mindfulness was not allowed to be the main topic of the course curriculum itself. Additionally, only participants (lecturers and students) older than 18 years, and capable to give informed consent were included. Lecturers recruited students during their first teaching session of the semester via verbal and written information about the study (e.g., study flyer). Participation was voluntary for both lecturers and students. At the end of the study, *n* = 30 students were randomly drawn from the post-sample and received a monetary reimbursement. Psychology and biomedical engineering students received study credits for participation.

### Procedure and intervention

After informing about the study, lecturers provided the links for a baseline survey (lasting approximately 15 min for students and 5–10 min for the lecturers). Completion took place until the next teaching session. In the following weeks, lecturers presented two conditions (course start with vs. without brief mindfulness exercise), in alternating order. The study design followed an ABAB design. Thus, all student participants received both conditions. We chose this design to ensure an easy implementation for lecturers despite varying durations between courses. The intervention consisted of a brief (3- to 4-min) mindful breathing space, as introduced in the MBCT curriculum^38^. The aim of the exercise is to first mindfully observe their own state of mind (all present thoughts, feelings, body sensations), second, observe their own breath and follow its movement through the body, and third, turn back mindfully to one’s own state of mind and potential changes in comparison to the beginning of the exercise.

Across conditions, students and lecturers completed an interim survey I (1 min.) before the regular course started (for the treatment condition: after the breathing space). At the end of each course, the students had the option to complete an interim survey II (1 min.) to re-assess their current mental state.

After the last teaching session of the semester, all participants were asked to complete a post-survey (again 15 min for students, 5–10 min for lecturers). All surveys were implemented in LimeSurvey and administered online.

### Measures

#### Primary outcome

Our primary outcomes were the current mental states of students and lecturers after the implementation of a brief mindfulness exercise at the beginning of the course, compared to no such exercise (interim survey I). Students received the same items again after each teaching session (interim survey II). At each interim survey, students and lecturers were asked to assess their current mental state with visual analogue scales ranging from *not at all* (0) to *completely* (100) with the following items: „How do you feel at this moment?” (a) “concentrated”, (b) “alert”, (c) “present”, (d) “distracted by thoughts”, (e) “energized”, (f) “stressed”. One extra item assessed current mood with a slightly different scale (g) “My mood is” *negative* (0) to *positive* (100). Only prior to the teaching session (interim survey I), students were also asked to rate their (h) “motivation for the upcoming teaching session”.

#### Secondary outcome

In the post-survey, all participants were asked to evaluate the intervention throughout the semester regarding its effect on their mental state with separate items asking for (a) “concentration”, (b) “alertness”, (c) “presence”, (d) “distraction by thoughts”, (e) “energy”, (f) “stress perception”, (g) “mood”, (h) “learning success”, (i) “contentment”, and (j) “closeness between participants within the course”. Lecturers were asked to answer these items for themselves and for the perceived effects on students’ mental states.

#### Control variables

At baseline- and post-survey, students received multiple questionnaires: To assess trait mindfulness, we used the *Freiburg Mindfulness Inventory* (FMI-14^40^; Cronbach’s α at baseline = 0.77), which includes 14 items, each presented with a 4-point response scale ranging from *rarely* (1) to *almost always* (4). A mean score was calculated after excluding the negatively phrased item (item 13) as recommended^41, 42^.

To assess personality dimensions, the brief 25-item version of the *Personality Inventory for DSM-5* (PID-5 BF^43^) was implemented. Responses are rated with a Likert scale ranging from *very false* (0) to *very true* (3). The PID-5 has five subscales (*Negative Affectivity*, *Antagonism*, *Disinhibition*, *Psychoticism*, and *Detachment*), all of which are calculated by building a mean score. In this study, we only focused on the Negative Affectivity subscale (Cronbach’s α at baseline = 0.64).

Additionally, we assessed psychological stress using the *Perceived Stress Scale* (PSS-10^44^; Cronbach’s α at baseline = 0.85). It includes 10 items, each rated on a 5-point response scale ranging from (*never)* 0 to *very often* (4). A sum score was calculated.

Further, to assess trait abilities in attentional control, we used the *Effortful Control subscale* (19 items) of the *Adult Temperament Questionnaire*, which contains a total of 77 items (ATQ^45, 46^). Internal consistency at baseline for the Effortful Control subscale was Cronbach’s α = 0.74. The items are assessed with a Likert scale ranging from *not at all applicable* (1) to *completely applicable* (7). We calculated a mean score for further analyses.

Finally, we assessed meditation experience at baseline as a control variable (“meditation frequency”, ranging from 0 = *not at all* to 100 = *a lot* and “experience” in years). Only lecturers were asked to rate their “experience in instructing meditation exercises” on a visual analogue scale ranging from *not at all* (0) to *a lot* (100).

To control for whether students actually participated in the entire 3- to 4-min exercises, we included four options for students to rate the offered “condition” in the interim survey I: (a) “The exercise was offered today, and I *fully* participated”, (b) “The exercise was offered, but I only participated *partly*”, (c) “The exercise was offered, but I did *not* participate” and (d) “There was no exercise offered today” (control condition). To further verify the students’ answers regarding the condition, we also asked lecturers whether they had offered the exercise or not.

Students also rated the following items: “teaching session held in person” vs. “online teaching session” vs. “the teaching session was recorded”, as well as camera “off” vs. “on” during the exercise, and quality of internet connection during exercise (“stable” vs. “not stable but did not bother me” and “not stable and it bothered me”).

The lecturers rated their experience during the exercise and their adherence to the instructions (“During the instruction, I …” (a) “felt well”, (b) “felt authentic”, (c) “was following the standardized instructions”) with a visual analogue scale, ranging from *not at all* (0) to *completely* (100).

### Data analyses

#### Power analysis and descriptive statistics

All analyses were done using R version 4.2.1^47^ and MPlus version 8.1^48^.

We used the summary-statistics-based power analysis to calculate the minimum sample size (https://koumurayama.shinyapps.io/summary_statistics_based_power/;^49^). As we are not aware of another study with a similar within-group design, we based the power analysis on the meta-analytic results of Dawson et al.^50^, who reported an effect size of *d* =  − 0.47 for the post-comparison of distress scores between mindfulness-based interventions and passive controls. The summary-statistics-based power analysis tool requires *t*-values, but because the *t*-distribution is almost identical to the *z*-distribution in large samples^51^, we used the reported Z-value (see Supplementary Fig. 1,^49^). Thus, based on* Z* = 7.06 and a level-2 sample size of *N* = 2201, the minimum sample size to achieve 80% power is *N* = 324. With an expected drop-out of approximately 30%, *N* = 200 * (1 ÷ 0.7) ≈ 462 student observations are needed. The data analysis of potential effects on the lecturers was exploratory, that is why no power estimation was done.

Next to our main outcome, we wanted to determine differences between pre- and post-measurements of students. To conduct an Intent-to-Treat analysis and thus account for missing data at post-measurement, we used regression analyses with full information likelihood estimation. This estimation method is implemented in the *sem()* function of the lavaan package^52^. The respective trait variables were used as outcomes and time as a dummy-coded predictor (pre = 0, post = 1). Effect sizes indicate a very small effect for all *d* < 0.1, a small effect for all 0.1 ≤ *d* < 0.3, and a moderate effect for all 0.3 ≤ *d* < 0.5^53^.

Spearman’s correlation coefficients were calculated to determine construct validity between primary and secondary variables as well as test–retest reliability (see Supplementary Information Figs. S1 and S2).

#### Data preparation

Our analysis differed slightly from our preregistered analysis plan regarding three aspects. First, instead of using eight different outcome variables, we aimed to use composite scores of the primary outcome items to reduce dimensions. Second, to probe our second hypothesis, we did not use change scores, but the mental state scores reported after the lecture (interim survey II). Third, instead of including every control variable step by step, we modelled a base model with all control variables, and added the predictor of interest (condition) in a second step.

To reduce dimensions, we applied exploratory factor analyses. As our data included multiple measurements per subject and condition, we followed the guidelines for multilevel exploratory factor analysis (for more details see explanation and Tables S1–S4 in the Supplementary Information^54^). Briefly, we computed an exploratory maximum likelihood factor analysis across all repeated measurements, conditions, and individuals, and two separate factor analyses for within-subject and between-subject variance. Finally, we performed a multi-level exploratory factor analysis using MPlus by computing the factor structure at each within-subject and between-subject level simultaneously. After examining all factor solutions carefully, we averaged the items “concentration”, “energy”, “presence”, and “alertness” to build factor 1 (presence composite score, Cronbach’s α = 0.90), and “stress” and “distraction by thoughts” to build factor 2 (stress composite score, Cronbach’s α = 0.66). Thus, the presence composite score refers to a state of being engaged in or focused on the present moment. The stress composite score reflects a state of being distracted or mentally absent and stressed in the current situation.

We used linear mixed-effects models to address the hierarchical structure of our data. All numeric predictors were scaled by two times the predictor’s standard deviation. All outcome variables were scaled by one standard deviation before entering into the models. According to^55^, this procedure allows to directly compare regression coefficients of continuous predictors and (untransformed) binary predictors. Models were visually checked for relevant model assumptions using the *check_model()* function^56^, which also includes the calculation of Cook’s distance. In contrast to the analysis plan in our preregistration, we did not conduct a sensitivity analysis in case of outliers, but decided to use a robust estimation method, which has the advantage that no observations need to be excluded. Therefore, in the case of influential cases, multicollinearity, non-normal distribution of residuals, or heteroscedasticity, we estimated the models again using the robust estimation method offered by the robustlmm R package^57^, which applies a Huber function aiming for more robust variance components and random effects. Here, we used default settings (computation method = "DAStau", *k* = 1.345, *s* = 10). Only if the robust and original models showed different results regarding the significance of predictors, we report the robust models rather than the original ones.

#### Primary outcome

To estimate the effect of the mindfulness exercise (vs. control condition), we used the observations from students who participated in the full 3- to 4-min mindful exercise. If not indicated otherwise, “participation” refers to *full* participation in one exercise (not during the whole semester). Please note that we excluded all student observations for which *no* or *partial* participation in one of the exercises was reported. However, these data are reported in the Supplementary Information (Table S6).

To test our first hypothesis (H1), we probed whether the condition (participation in the mindfulness exercise vs. control, fixed effect) predicted students’ mental states at the beginning of the teaching session. The (a) presence composite score, (b) stress composite score, (c) mood item, and (d) motivation for the lecture item served as outcome variables in four distinct models. Some students attended more than one course, which is why subjects and courses were partially crossed and not nested factors^58^. Using the lme4 package^58^, it is possible to address partial crossing by modelling two random intercepts. Therefore, we added random intercepts for subject IDs and course IDs in a first model. Additionally, since all participants received both conditions in alternating order, in a second step, we added random slopes for “condition” (varying across subjects and courses). The second model revealed boundary issues indicating that there were too few observations given the model complexity. Consequently, the simple random intercepts model was kept as the final model. This strategy was used for all four outcome variables. All models were controlled for the variables age, gender, meditation experience, meditation frequency, and modality of the lecture (online vs. in-person).

To probe the second hypothesis (H2) stating that the described effects of the mindful exercise condition last throughout the teaching session, we fitted three random intercept models with the (a) presence composite score II, (b) stress composite score II, and (c) mood item II as outcome variables (using the data from interim survey II *after* the teaching session), condition (participation vs. control) as the independent variable, and subject IDs, as well as course IDs as random intercepts. Also, the same control variables were included as in the analysis of the first hypothesis.

#### Secondary outcome

To address our exploratory research questions (secondary outcomes), we added several control variables to our base models (see H1):

First, to probe whether baseline variables moderate the effect of the mindfulness exercise on the mental state of students (E1), we added different interaction terms to our base models (Baseline variable × Condition). More specifically, we modelled (a) the effect of Psychological Stress × Condition on the stress composite score, (b) the effect of trait Attentional Control × Condition on the presence composite score, and (c) the effect of Negative Affectivity × Condition on mood before the course. In each of the models, the effect of trait Mindfulness × Condition was included as well as age, gender, meditation frequency, meditation experience at baseline and modality of the lecture (online vs. in-person).

Second, we aimed to address the potential effects of lecturers’ characteristics on students’ mental states (E2). Here, we dropped all observations of the control condition because lecturers rated these items only after instructing the mindfulness exercise. Thus, all four outcome variables were predicted by the (time-varying) lecturers’ reports of their level of (a) well-being and (b) authenticity during the instruction, as well as the (c) standardization of the instruction.

Third, because our sample of lecturers was relatively small, we refrained from using the same methods as for the student sample, and instead opted to report all lecturers’ data on a descriptive level only (E3).

Finally, next to a descriptive analysis of the evaluation (E4), we investigated which factors influenced students’ retrospective overall evaluation of the intervention at the end of the semester (subjectively rated effects on different aspects of learning and well-being). We calculated a composite score for all ten evaluation items, which we then used as an outcome variable. Participation time per condition (up to six times per control condition or full participation in the mindfulness exercise), setting (“online courses” vs. “courses held in person”), and the individual (time-varying) presence composite score, stress composite score, mood, and motivation for the courses served as predictors in this last model (E5).

Further analyses examining Condition × Time interaction effects, effects of lecturers’ experience in meditation, and effects of setting modalities of the courses on the outcome variables are reported in the Supplementary Information (see Table S6).

## Results

### Descriptive statistics

**Figure 1:**
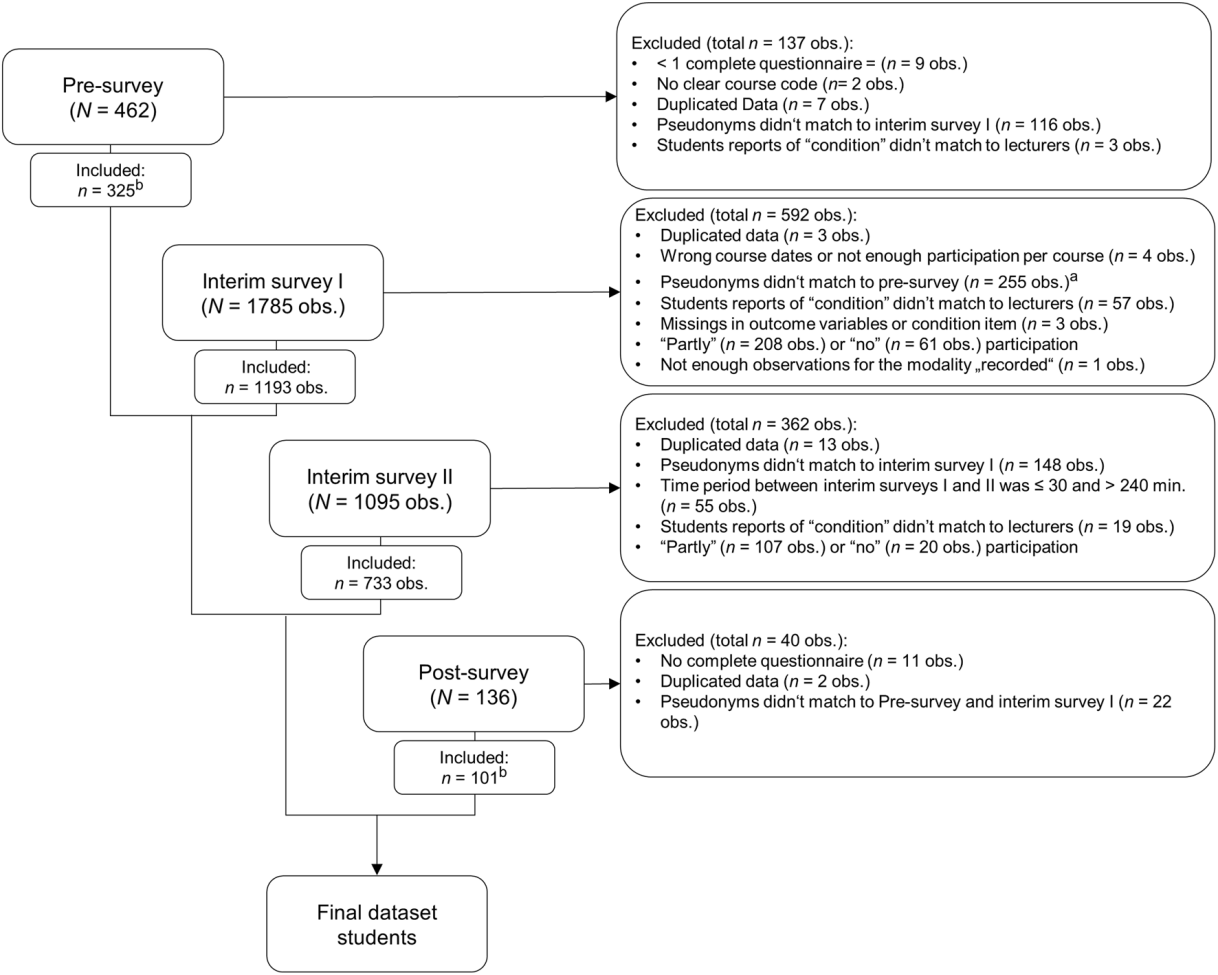
Flow chart of student participants. ^a^We changed misspellings in *n* = 18 observations while merging pre-survey and interim survey I data via similarity (1 − jacchard index, similarity ≥ .75) and visual similarity (only those with the same course number, and only one letter missed or confused). In using this method, we could save *n* = 67 observations of the interim survey I that found a match in pre-survey, and *n* = 16 observations of pre-survey that found a match in interim survey I. Additionally, we corrected *n* = 4 misspellings in course codes. ^b^Since in the pre-survey *n* = 11 and in the post-survey *n* = 5 students had missing values in relevant questionnaires, we excluded these students from relevant subanalyses.

We excluded several observations due to missing data, incorrect course codes, or non-matching participant codes between pre-survey, interim surveys, and post-survey (see Fig. 1; for a flow chart of lecturers’ data see Fig. S3 in the Supplementary Information). We exceeded the calculated sample size with *n* = 600 observations that were collected during the control condition, compared to observations of students that fully (*n* = 593) or partly (*n* = 208) participated, or did not participate (*n* = 61) in the mindful exercise.

In total *N* = 325 students (see Table 1 for student characteristics at baseline and post-measurement) and *N* = 14 lecturers were included in the analysis (*M*_*age*_ = 47.2, *SD*_*age*_ = 9.4 years; 36% female, *n* = 10 lecturers at post-measurement). Students were recruited from *N* = 19 courses in different areas (information technology, business administration, psychology, electronic measurements, structural mechanics, food technology, biomedical engineering, health communication and applied ethics). Frequency of meditation increased over time (*Z* = 2.83, *p* = 0.005, *d* = 0.11; see Table 1). There was no difference between pre- and post-measurements for negative affectivity (*Z* = 1.25, *p* = 0.210, *d* = 0.05), attentional control (*Z* = 0.63, *p* = 0.528, *d* = 0.02), stress (*Z* = − 1.13, *p* = 0.260, *d* = -0.04), and trait mindfulness (*Z* = 1.78, *p* = 0.076, *d* = 0.27).

**Table 1:** Student characteristics at baseline- and post-measurement.

	Pre (*N* = 325)^a^	Post (*n* = 101)^a^	*z*	Cohen's *d*
Age	22.9 (4.6)	23.0 (3.8)		
Gender				
Non-binary	1 (0.3%)	–		
Female	197 (61%)	72 (71%)		
Male	127 (39%)	29 (29%)		
Meditation experience [years]	1.1 (2.1)	–		
FMI-14^b^	2.6 (0.4)	2.7 (0.4)	1.78	0.07
ATQ effortful control	4.4 (0.7)	4.4 (0.7)	0.63	0.02
Missing observations	3	3		
PID-5 Negative affectivity	1.3 (0.6)	1.4 (0.6)	1.25	0.05
Missing observations	11	5		
PSS-10	19.0 (15.0, 24.0)	18.0 (14.0, 23.0)	− 1.13	− 0.04
Meditation frequency [VAS]	6.3 (0.0, 25.2)	16.7 (0.7, 40.1)	**2.83**	**0.11**

*FMI* Freiburg Mindfulness Inventory, *ATQ* Adult Temperament Questionnaire, *PID* Personality Inventory for DSM-5; *PSS* Perceived Stress Scale, *VAS* Visual Analogue Scale (0 = *not at all* to 100 = *a lot*), *df* degrees of freedom.

Significant values in bold.

^a^Mean (SD); n (%); Median (IQR = interquartile range).

^b^Mean score included only 13 items.

Of the *N* = 14 lecturers, 50% attended all three coaching appointments, 30% attended two, and 20% only one. The average meditation experience of the lecturers was 8.6 years (*SD* = 11.3, Range = 1–40). The reported meditation frequency (*M* = 59.1, *SD* = 26.6) and the experience in instructing (*M* = 47.3, *SD* = 37.6) were both rated at a medium range on a visual analogue scale ranging from *not at all* (0) to *a lot* (100).

Across both conditions (participation vs. control), we were able to include *n* = 1193 observations of students for interim survey I (excluding no or partial participation in one of the exercises), *n* = 733 observations for interim survey II, and *n* = 160 observations of lecturers over the course of the semester. Most courses were held online due to the ongoing COVID-19 pandemic (*n* = 17 of 19 courses). Of the two courses held in person, *n* = 67 student observations were collected. Regarding the reports of lecturers, *n* = 94 observations of lecturers were collected during the mindfulness condition and *n* = 72 observations during control condition. Students could participate up to six times in the mindful exercise and up to five times in the control condition with an average participation per condition of *M* = 2.09 (*SD* = 1.21). Because not all courses were offered a maximum of twelve times, decreases in students’ response rates over the course of the semester may be due to reduced course appointments and must be interpreted with caution (see Supplementary Information, Table S5).

### Primary outcome

#### Effects on students’ mental states at the beginning of teaching sessions

**Figure 2:**
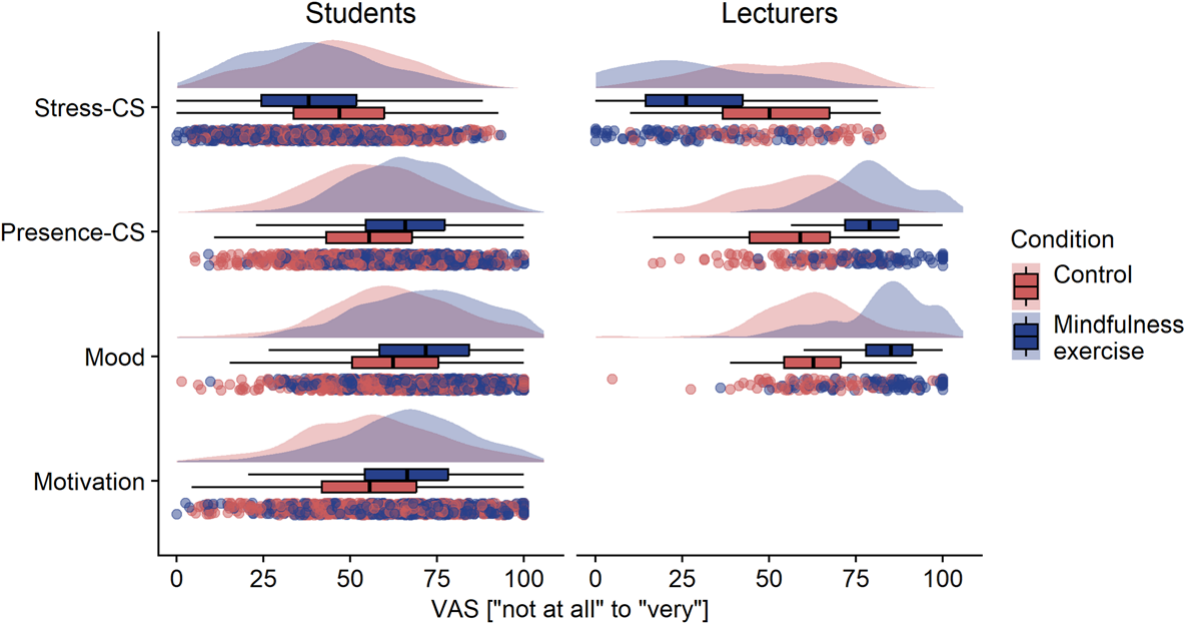
Students’ and lecturers’ reports of their mental states after control condition vs. mindfulness exercise. Stress-CS = stress composite score; Presence-CS = presence composite score; Raincloud plots are displayed that include split-half violin plots showing the distribution of data, boxplots, and raw jittered data.

Figure 2 shows the median of the four outcome variables (a) presence composite score, (b) stress composite score, (c) mood, and (d) motivation for the course separated per condition (H1). According to the multilevel models, participation in the mindful exercise yielded significantly higher current presence, lower stress, better mood, and higher motivation for the lecture compared to the control condition without mindful exercise (see Table 2). The fixed effects alone (participation vs. control) explained very little variance^53^. However, the conditional *R*^2^ indicated that fixed and random effects together explained a substantial proportion of variance^53^. Moreover, variance between courses (Range τ00_course_ = 0.00–0.08) was rather low compared to variance between (Range τ00_subjects_ = 0.36–0.46) and within subjects (Range σ^2^ = 0.47–0.56). Therefore, a high conditional *R*^2^ implies that variance in the outcome variable was mainly explained by between- and within-subject variances (random effects), rather than by the mindfulness exercise (fixed effect) alone.

**Table 2:** Effects of mindfulness exercise on students’ mental states before and after the teaching session and in interaction with baseline variables.

	Outcome variables															
	Presence composite score				Stress composite score				Mood				Motivation			
	*ß* 95% CI		*p*	*R*^2^*/*cond.* R*^*2*^	*ß*	95% CI	*p*	*R*^2^*/*cond.* R*^*2*^	*ß*	95% CI	*p*	*R*^2^*/*cond.* R*^*2*^	*ß*	95% CI	*p*	*R*^2^*/*cond.* R*^*2*^
H1: Effect of mindfulness exercise *before* the teachings session (*n* = 1193 obs.)																
				0.08/0.46				0.05/0.45				0.06/0.53^a^				0.06/0.52^a^
(Intercept)	0.32	− 0.73, 0.08	0.119		**0.53**	0.11, 0.95	**0.014**		− 0.20	− 0.59, 0.20	0.328		0.02	− 0.44, 0.48	0.934	
Condition (mindfulness exercise)	**0.55**	0.46, 0.64	** < 0.001**		− **0.37**	− 0.46, − 0.28	** < 0.001**		**0.38**	0.30, 0.47	** < 0.001**		**0.43**	0.34, 0.52	** < 0.001**	

H2: Effect of mindfulness exercise *after* the teaching session (*n* = 733 obs.)																
				0.03/0.41				0.03/0.41				0.04/0.42^a^				
(Intercept)	0.03	− 0.62, 0.67	0.938		0.28	− 0.39, 0.94	0.410		− 0.15	− 0.76, 0.47	0.643					
Condition (mindfulness exercise)	**0.29**	0.17, 0.41	** < 0.001**		− **0.19**	− 0.31, − 0.07	**0.002**		**0.20**	0.08, 0.31	**0.001**					

E1: Effect of mindfulness exercise *before* the teaching session in interaction with baseline variables (*n* = 1165–1193)^b^																
				0.13/0.47				0.12/0.46				0.13/0.49				0.09/0.53
(Intercept)	− 0.25	− 0.65, 0.15	0.218		**0.43**	0.02, 0.84	**0.039**		− 0.06	− 0.46, 0.33	0.746		0.05	− 0.41, 0.51	0.822	
Condition (mindfulness exercise)	**0.55**	0.46, 0.64	** < 0.001**		− **0.37**	− 0.46, − 0.28	** < 0.001**		**0.37**	0.29, 0.46	** < 0.001**		**0.43**	0.34, 0.51	** < 0.001**	
Mindfulness	**0.33**	0.13, 0.52	**0.001**		− **0.24**	− 0.45, − 0.02	**0.029**		**0.39**	0.18, 0.61	** < 0.001**		**0.40**	0.21, 0.60	** < 0.001**	
Mindfulness × condition (participation)	0.04	− 0.14, 0.22	0.640		0.03	− 0.16, 0.23	0.725		0.04	− 0.15, 0.23	0.666		0.01	− 0.16, 0.18	0.876	
Attentional control	**0.26**	0.06, 0.45	**0.009**													
Attentional control × condition (mindfulness exercise)	− 0.01	− 0.19, 0.17	0.935													
Stress					**0.42**	0.20, 0.63	** < 0.001**									
Stress × condition (mindfulness exercise)					0.03	− 0.16, 0.22	0.759									
Negative affectivity									− **0.31**	− 0.53, − 0.09	**0.006**					
Negative affectivity × condition (mindfulness exercise)									0.06	− 0.13, 0.24	0.566					

All models displayed are controlled for age, gender, experience in meditation at baseline, frequency in meditation at baseline, and lecture modality (online vs. in-person lecture). Due to conciseness, we refrained from displaying beta estimates of control variables; ß = standardized regression coefficient.

Significant values in bold.

^a^Robust model is displayed.

^b^Sample size varied between models because of missing values for respective self-report data at baseline.

Results including observations of students that did not or only partially participate in one of the exercises were in line with the current pattern (see Supplementary Information, Table S6). Also, only in the supplemental analysis of incomplete data without the control condition, the motivation for the exercise was an important predictor for our outcome variables (see Supplementary Information, Table S6).

#### Effects on students’ mental state after the teaching sessions

The effect of participation in the mindfulness exercise lasted throughout the teaching session for all three outcome variables (presence composite score II, stress composite score II, mood II) compared to control teaching sessions without offering such exercises (H2; see Table 2). Here, *n* = 733 observations were included in the four models. Marginal *R*^*2*^ for each of the models was very small^53^. Conditional *R*^2^ indicated that the fixed and random effects explained a substantial proportion of variance^53^.

### Secondary outcome

#### Effect of trait variables

We did not find interaction effects of the condition with different baseline variables (E1; see Table 2). Thus, irrespective of personality traits, stress at baseline, and abilities of attentional control, students exhibited positive mental effects from participating in the mindfulness exercise compared to no such exercise. All four models were controlled for age, gender, meditation frequency, and meditation experience at baseline as well as for the modality of the lecture.

Nevertheless, we found different main effects showing that higher attentional control at baseline was associated with higher levels in the presence composite score. Higher stress at baseline was a significant predictor for higher levels in the stress composite score, and higher levels in the personality trait negative affectivity were associated with lower mood levels. Also, higher trait mindfulness predicted higher presence, lower stress levels, better mood, as well as higher motivation for the teaching session at the beginning of a teaching session (see Table 2).

#### Effects of lecturers’ characteristics

To investigate the effect of the situational items “well-being”, “authenticity” and “following the standardized instructions” assessed in lecturers during the instruction (E2), we dropped all observations of the control condition, because lecturers rated these items only after instructing the mindfulness exercise. In total, *n* = 578 student observations were included in these models. Here, no effects of lecturers’ characteristics on our four outcomes in the students were found (all *p*s > 0.05).

#### Effects on lecturers

On a descriptive level lecturers reported higher levels of energy, alertness, presence, and concentration (combined in a presence composite score), lower distraction and stress levels (combined in a stress composite score), as well as better mood when instructing the mindfulness exercise compared to teaching sessions without such exercise (see Fig. 1).

#### Evaluation of the exercise effects at post-measurement

**Figure 3:**
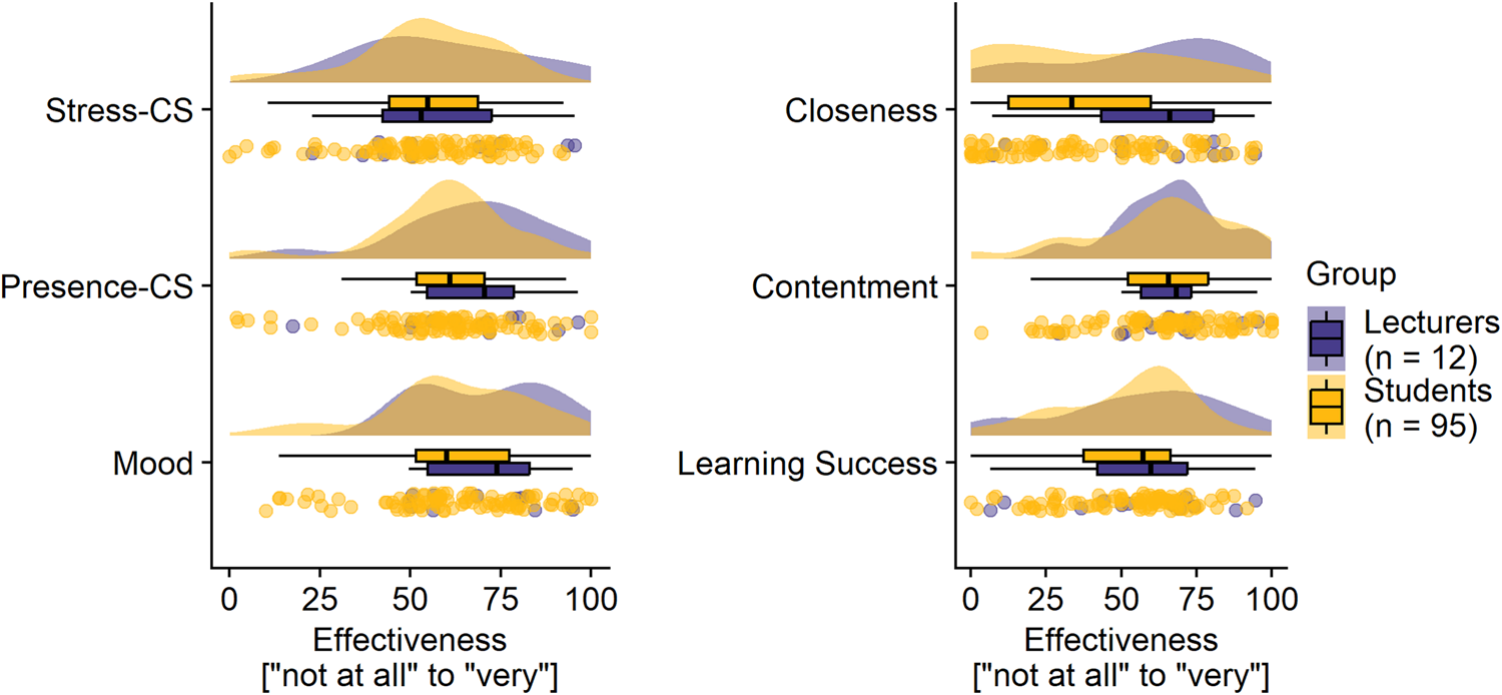
Students’ and lecturers’ evaluation at the end of the semester regarding the effect of the mindfulness exercise on different facets of learning and teaching. Stress-CS = Stress composite score; Presence-CS = Presence composite score; Raincloud plots are displayed that include split-half violin plots showing the distribution of data, boxplots, and raw jittered data.

Figure 3 displays raincloud plots for the effects on different aspects of students’ well-being (e.g., presence composite score, stress composite score, mood) and learning success subjectively rated by both students and lecturers at the end of the semester. In general, lecturers estimated the effects of the brief mindfulness exercise on the student outcomes to be the same or greater than the students. On a descriptive level, the mean ratings of students and lecturers differed for the item “closeness between course participants”, whereby lecturers rated the effect on closeness higher than the students did. Regarding all other aspects, the effects of the mindfulness exercise were rated by lecturers and students in a medium range.

#### Overall evaluation

We also identified factors that influenced students’ overall evaluation of the intervention at the end of the semester (marginal *R*^2^ = 0.10; conditional *R*^2^ = 0.31). Here, a higher overall evaluation composite score was predicted by higher situational presence before the teaching session (*ß* = 0.48, 95% CI [0.27, 0.69], *p* < 0.001) and higher situational motivation for the course (*ß* = 0.24, 95% CI [0.06, 0.43], *p* = 0.011), but not by mood levels (*ß* = − 0.02, 95% CI [− 0.24, 0.20], *p* = 0.841), stress composite score (*ß* = 0.10, 95% CI [− 0.06, 0.26], *p* = 0.217), number of times of participation per condition (*ß* = 0.03, 95% CI [− 0.02, 0.09], *p* = 0.252), or course modality such as “online courses” vs. “courses held in person” (*ß* = − 0.00, 95% CI [− 0.63, 0.63], *p* = 0.998).

## Discussion

In the current study, we investigated the feasibility of implementing a brief mindfulness exercise at the beginning of regular university courses. In a within-subject design, we also compared the effects of this brief exercise to the normal status quo, that is, starting the course without an exercise. More specifically, we probed whether a 3- to 4-min mindful breathing space had an immediate positive effect on students’ mental states and whether said positive effect persisted throughout the teaching session.

We also aimed to explore whether the effect of the mindfulness exercise on students’ mental state depended on the different student- and lecturer-related trait and state variables. Last, we asked how students and lecturers assessed the overall long-term effect of the intervention at the end of the semester.

### Primary outcome

Our results show that the participation in a brief mindfulness exercise at the beginning of each session of a university course has immediate positive effects on the mental states of students, resulting in higher presence, lower stress, better mood, and higher motivation for the course compared to starting the course with no such exercise, as is the current status quo.

These results are in line with other studies showing beneficial effects of brief mindfulness trainings on students’ mental health (^9^; for a meta-analysis on negative affectivity see^36^). Also, in accordance with other studies (investigating mindfulness-based trainings with a higher dose; for meta-analyses see^18, 50^), we were able to show that a very brief mindfulness exercise already has a stress-reducing effect. Since most of these studies refer to self-guided programs or weekly trainings that are independent of the actual university courses, our findings go beyond previous reports. They highlight that the particularly cost- and time-efficient implementation of a mindfulness exercise within the teaching session positively affects students’ current mental well-being. Moreover, and as hypothesized, these positive effects persisted throughout the teaching session, such that students exhibited in combination, higher levels of presence, alertness, energy, and concentration (presence composite score), lower stress and distraction by thoughts (stress composite score), and better mood at the end of a session.

By fostering a positive mental state as well as the motivation of students, necessary prerequisites for successful academic learning are set: The importance of motivational processes for learning and performance, in general, has been studied for decades^59, 60^. Learning motivation has been negatively linked to academic burnout^61^ and different aspects of inattention^35^, as well as positively to learning engagement^61^. Lower motivation has also been associated with more mind wandering, more external distractions, and a lower ability to focus in online lectures during the COVID-19 pandemic^35^. Additionally, particularly positive compared to negative emotions have been associated more strongly with motivation^62^.

Cultivating positive emotions and motivation could therefore not only positively affect student’s performance, but also trigger a cascade of steps leading to positive reinforcement, lower stress, and more engagement and attention (and thus less mind wandering). Because mind wandering is also associated with poorer academic performance^63, 64^ and strongly overlaps with facets of the stress and presence composite scores we measured (i.e., distraction by thoughts and lower presence), the mindfulness exercise may have actually improved learning performance by reducing distraction and strengthening presence. However, mindfulness practice may not be a universal panacea for everyone: A recent large-scale universal prevention program implemented in schools did not show generalized positive effects on depression symptoms and well-being (MYRIAD study^65, 66^). Nevertheless, it must be noted that these results are not directly comparable to adult samples, as meta-analyses did show positive effects of mindfulness interventions for adults^10^.

In summary, lecturers were able to integrate a brief mindfulness exercise into their teaching curricula, which yielded positive effects on students’ mental states, such as higher presence, energy, alertness, less stress, improved mood as well as higher motivation for the course. Although academic performance and learning capacities were not directly assessed, there is good evidence in the literature that an improvement in the targeted mental states is beneficial for successful academic engagement, learning, and performance in the long run.

### Secondary outcome

Next to the general effect of the brief mindfulness exercise, we were also interested in exploring which specific variables moderate the said effects on the mental states of students.

#### Effect of trait variables

Interestingly, there were no interaction effects of different baseline trait variables with the mindfulness exercise, indicating that mindfulness, negative affectivity, or stress at baseline did *not* strengthen or weaken the effect of the mindfulness exercise. Rather, trait mindfulness seemed to have generally positive effects on presence, stress, mood, and motivation for the courses, irrespective of the displayed condition. Also, high negative affectivity (which is closely linked to neuroticism^67^) was predictive of a more negative mood. Higher trait attentional control was predictive of higher situational presence in the courses. Moreover, high psychological stress at baseline was associated with high levels of acute stress.

Although personality traits have been associated with (trait) mindfulness in multiple correlational and cross-sectional studies (for meta-analysis see^25^), research investigating moderating effects of personality traits on mental health outcomes following a mindfulness-based intervention is rather scarce and has yielded inconclusive results. In line with our findings, in one randomized controlled trial, trait mindfulness was not a significant moderator of the effects of MBSR-training on different mental health outcomes^68^. Another study found no interaction of trait mindfulness and time on stress, although this result is not directly comparable, because no control condition was realized in the study^69^. Contrasting our results, other studies found that neuroticism moderated an intervention effect on mental health and subjective well-being^68, 70^: Individuals with higher levels of neuroticism seemed to benefit more from a mindfulness-based intervention compared to those with lower levels. As the intervention dose in these studies was much higher than in ours, a modulatory effect of personality traits may have been masked in our study.

Regarding attentional processes, we found that next to the mindfulness exercise, trait attentional control significantly predicted situational presence measured by the composite score of presence, energy, concentration, and alertness item. The absence of an interaction effect indicates that higher trait attentional control did not increase the effect of our intervention on situational presence. Other studies investigating attentional control and mindfulness did not explore interaction effects but looked more broadly at the malleability of performance in attentional control tasks via mindfulness training and came to inconclusive results: Whereas some studies found an increase in attentional control after a mindfulness training^71, 72^, other studies did not find such evidence for attentional processes (for meta-analysis see^17^). Note that compared to these studies, we investigated the effects on *situational* presence. Also, measures of attentional control vary largely between studies. Comparisons must therefore be made with caution. Nevertheless, future studies should look more deeply into the moderating role of attentional control on the effects of mindfulness interventions.

With respect to participants’ stress load, we did not find that perceived stress at baseline moderated the effect of the mindfulness interventions on situational feelings of stress and distraction by thoughts. Similiar to the above-mentioned null findings, this result may be explained by the dose effects of our intervention.

Altogether, our results provide important insights into the question of who benefits most from a brief mindfulness exercise. Interestingly, our results suggest that positive training effects are independent of the assessed traits, suggesting a broad efficacy of the training. As the current results are exploratory, future studies may generate new hypotheses based on our findings.

#### Effects of lecturers’ characteristics

To our knowledge, this is one of the first studies investigating the association of lecturers’ state characteristics with students’ mental states after instructing a mindfulness exercise. None of the lecturers’ characteristics had a significant effect on the mental states of students. Rather, under careful consideration of the small sample of lecturers included in this analysis, our results seem to suggest that, regardless of the authenticity or well-being of the instructor during the instruction, the mindfulness exercise has positive effects. In contrast, prior research highlights the importance of embodiment as a “key feature” for teaching mindfulness^73^. We assume that in longer and more engaged mindfulness interventions, lecturers’ characteristics may have a more prominent influence on the practice of their students. Our results must be replicated with a larger sample of lecturers. However, they may be of particular interest to and encouraging for new mindfulness instructors who have not yet reached a level of practice in which they are embodying their mindfulness instructions.

#### Effects on lecturers

Teaching mindfulness requires awareness of students’ responses as well as of one’s own presence during the instruction^73^. Thus, teaching mindfulness goes along with higher demands than simple practice for one’s own benefit. Our descriptive analyses of the lecturer’s data implied that despite such increased demands, lecturers nevertheless reported higher presence, energy, alertness, concentration, less stress and distraction, and better mood compared to those weeks without a mindful course start. These descriptive results do not allow final conclusions but may serve as basis for future confirmatory research.

#### Evaluation

At the end of the semester, both students and lecturers positively evaluated the intervention. Students that reported higher situational motivation for the lecture and higher levels in the presence composite score (averaged across both conditions) also rated the overall effect of the mindfulness exercises higher. These findings highlight, again, how important motivational processes are for learning and teaching, but also for participating in and benefitting from interventions. Since we did not ask students why they declined participation, our results over-represent the perception of students who were motivated. Nevertheless, our study was constructed as a feasibility and pilot study, and the evaluation results show that the implementation of a brief mindfulness exercise does reach students, and that interested students do have beneficial effects.

### Limitations

We must acknowledge several limitations of our study. First, the response rates of students decreased substantially towards the end of the semester. Since students did not receive reimbursement (except for the “lottery” winners), this is not surprising and is in line with other online or self-help studies showing high drop-out rates^9^. However, we did not ask lecturers how many students participated per course. Therefore, it is impossible to differentiate whether students were less inclined to participate in the exercise and surveys, or rather failed to attend classes altogether. Also, we did not assess reasons for non-participations (e.g., lack of interest or late arrival to class) or whether students attended hybrid courses (online and in-person). Another limitation of our study design is that it does not allow independent between-group comparisons. A recent review of meta-analyses showed that when comparing mindfulness-based interventions to active control conditions within student samples, effects do not differ^12^. Since we did not implement an active control condition, it remains open whether the effect of our brief intervention is larger than other active interventions (e.g., relaxation exercises). Another limitation concerns our sample size calculation. We based our power analysis on reported effects of between-group differences from randomized controlled trials^50^, as we were not aware of any studies that used a design similar to ours. These effects are therefore not directly comparable to our within-group study design, in which all students received both conditions in an alternating order. Moreover, we only used self-reports. As the recruited lecturers were generally open to and interested in mindfulness, we cannot rule out that the expectancies of lecturers biased outcomes of students (*Rosenthal-effect*^74^). There also might be a general influence of expectancy effects on self-ratings. Last, it needs to be noted that our predictors only explained very little variance. This might be due to the longitudinal design and the time-varying outcome variables.

## Conclusion

Altogether, this preregistered multicenter study provided first evidence for the positive effects of a brief 3- to 4-min mindfulness exercise at the beginning of regular teaching sessions on students’ well-being and mental states, both immediately after the exercise and after the termination of each lecture. Students showed higher presence, lower stress, better mood, and higher motivation for the courses after participating in the mindfulness exercises compared to control weeks. No specific student traits influenced the benefit of the intervention. In summary, with the mentioned limitations in mind, our study showed that students and lecturers from different faculties were generally willing to accept and profit from a low-intensity mindfulness offer in the university setting.

## Supplementary Information

Supplementary Information.

## Data Availability

The study was preregistered at open science framework (https://osf.io/nxucw). Data and R scripts will be available at (https://osf.io/23b8w/?view_only=2e32d0049f614e9e9aff742b25df69a4).
