# Supplementary material for: A multicenter feasibility study on implementing a brief mindful breathing exercise into regular university courses
Source: Sci Rep. 2023 May 16;13:7908. doi: 10.1038/s41598-023-34737-0 (PMC10186318; doi:10.1038/s41598-023-34737-0)
Supplement: Supplementary file 1 — Supplementary Information. [file 41598_2023_34737_MOESM1_ESM.docx]

A multicenter feasibility study on implementing a brief mindful breathing exercise into regular university courses

Supplementary Information

# Exploratory Factor Analyses

In order to reduce dimensions, we aimed to use sum scores of the outcome items by applying exploratory factor analyses. We only used students’ observations assessed after participation in the mindfulness exercise and in the control condition (*n* = 1194 obs., interim survey I). We excluded all reports of “no” and “partial” participation in one of the exercises.

In a first step we checked how many factors should be extracted using the *n_factors()* function of the parameters package [1], which runs multiple factor extraction methods and gives the consensus between those methods. For all eight outcome items, 31.58% of the methods (6 out of 19) suggested just one dimension. Since one dimension would not have been a benefit for our analysis, and after content considerations regarding the “mood” and “motivation” items (linear combination of these two items with other items would have been difficult to interpret, motivation was only measured within interim survey I), we decided to remove these two items from factor analysis. For the remaining six items (“concentration”, “energy”, “alertness”, “presence”, “distraction by thoughts”, and “stress”) two dimensions were suggested by 43.75% of the methods (7 out of 16; e.g., Parallel analysis, Kaiser’s criterion, SE Scree). Since our data included multiple measurements per subject and condition, we followed the guidelines for applying a multilevel exploratory factor analysis [2].

For all exploratory factor analyses, we used maximum likelihood estimation method and oblique rotation (Geomin method). First, we conducted a single exploratory maximum likelihood factor analysis (across all repeated measurements, conditions, and individuals). Second, we computed intraclass correlation coefficients (ICCs) per item for each level in order to explore whether multilevel EFA was indicated (see Table S1). Since our data is partially crossed (some individuals visited more than one course), we used two random intercepts as recommended for course ID and subjects ID [3]. On the subject level, ICCs were on average larger than 0.1 [4], indicating high variability on this level and, thus, multilevel EFA seemed to be appropriate. On the course level, ICCs were lower than 0.1. We decided to compute distinct EFAs separating within-subject variance in step three and four (aggregated for condition plus repeated measurement variance) and the between-subject variance. Here, we used *demean()* function of the datawizard package [5] and computed between- and within-subject variance per item. Outcomes of the separate EFAs supported the suggested structure of the single EFA (see Table S2).

Finally, we conducted a two-level EFA using MPlus. Here, factor structure at each within-subject and between-subject level was computed simultaneously within one factor analysis (see Table S3). Although, within the two-level EFA, there were cross loadings at the within-subject level regarding the two items “concentration” and “presence”, we nevertheless decided to calculate sum scores based on the suggested factor solution, since within step three (EFA for within-subject variance) and four (EFA for between-subject variance) factor solutions did not differ. Thus, we averaged the items “concentration”, “energy”, “presence”, and “alertness” for factor 1 (“presence composite score”) and “distress” and “distraction by thoughts” for factor 2 (“stress composite score”).

## Intraclass correlation coefficients

| Table S1  *Intraclass Correlation Coefficients per Item on Subject and Course Level* | | | | | | | | |
| --- | --- | --- | --- | --- | --- | --- | --- | --- |
| Group | **distress** | **distraction** | **concentration** | **energy** | **presence** | **alertness** | ***M*** | ***SD*** |
| Subject ID | 0.38 | 0.28 | 0.31 | 0.32 | 0.29 | 0.31 | 0.31 | 0.03 |
| Course ID | 0.05 | 0.01 | 0.01 | 0.03 | 0.02 | 0.03 | 0.02 | 0.01 |

## Exploratory factor analysis for within- and between-subject variances and across all participants

| Table S2  *Factor Loadings and Uniqueness for Different Exploratory Factor Analyses using R* | | | | | | | | | | | | | | | |  |
| --- | --- | --- | --- | --- | --- | --- | --- | --- | --- | --- | --- | --- | --- | --- | --- | --- |
|  | **Across all subjects and conditions** | | | **Within-subject variance** | | | **Between-subject variance** | | | **Only control condition** | | | **Only mindfulness condition** | | |  |
|  | 1 | 2 | *u²* | 1 | 2 | *u²* | 1 | 2 | *u²* | 1 | 2 | *u²* | 1 | 2 | *u²* | |
| distress | 0.03 | **0.64** | 0.61 | 0.02 | **0.50** | 0.76 | 0.04 | **0.73** | 0.48 | 0.10 | **0.59** | 0.68 | -0.05 | **0.65** | 0.56 | |
| distraction | -0.05 | **0.73** | 0.45 | -0.01 | **0.63** | 0.60 | -0.07 | **0.79** | 0.34 | -0.10 | **0.70** | 0.46 | 0.01 | **0.80** | 0.37 | |
| concentration | **0.75** | -0.22 | 0.27 | **0.60** | -0.37 | 0.31 | **0.81** | -0.18 | 0.22 | **0.76** | -0.19 | 0.29 | **0.74** | -0.20 | 0.30 | |
| energy | **0.82** | 0.06 | 0.36 | **0.80** | 0.04 | 0.39 | **0.85** | 0.06 | 0.32 | **0.82** | 0.04 | 0.34 | **0.77** | 0.09 | 0.44 | |
| presence | **0.76** | -0.24 | 0.23 | **0.60** | -0.39 | 0.28 | **0.83** | -0.18 | 0.17 | **0.75** | -0.23 | 0.28 | **0.79** | -0.19 | 0.23 | |
| alertness | **0.88** | 0.08 | 0.28 | **0.83** | 0.03 | 0.33 | **0.92** | 0.11 | 0.21 | **0.91** | 0.08 | 0.22 | **0.84** | 0.08 | 0.34 | |
| Cronbach’s *α* | 0.90 | 0.66 |  | 0.87 | 0.57 |  | 0.92 | 0.74 |  | 0.90 | 0.59 |  | 0.88 | 0.71 |  | |
| *Notes.* *u²* = Uniqueness | | | | | | | | | | | | | | | | |

## Two-level exploratory factor analysis

| Table S3  *Factor Loadings of Two-Level Exploratory Factor Analysis using MPlus* | | | | | | |
| --- | --- | --- | --- | --- | --- | --- |
|  | **Within-subject** | | | **Between-subject** | | |
|  | 1 | 2 | Estimated residual variances | 1 | 2 | Estimated residual variances |
| distress | **0.48** | 0.03 | 0.79 | **0.81** | 0.09 | 0.39 |
| distraction | **0.57** | 0.00 | 0.68 | **0.99** | -0.01 | 0.02 |
| concentration | **-0.49** | **0.49** | 0.29 | -0.09 | **0.89** | 0.15 |
| energy | -0.00 | **0.79** | 0.38 | 0.01 | **0.87** | 0.25 |
| presence | **-0.50** | **0.50** | 0.27 | -0.09 | **0.94** | 0.06 |
| alertness | 0.01 | **0.83** | 0.31 | 0.10 | **0.98** | 0.10 |
| *Note.* Estimated residual variances (MPlus) may be not directly comparable to uniqueness estimated via R | | | | | | |

### Fit Indices

| Table S4  *Comparison of Fit Indices of all Exploratory Factor Analysis using R and MPlus* | | | | | | | | | |
| --- | --- | --- | --- | --- | --- | --- | --- | --- | --- |
|  | ***n* (obs.)** | ***Χ²*** | **df** | ***p*** | **RMSEA** | **TLI** | **RMSR** | **RMSR**  **(df corr.)** | **BIC** |
| EFA – across | 1194 | 102.32 | 4 | < 0.001 | 0.14 | 0.90 | 0.03 | 0.05 | 73.98 |
| EFA – within | 1194 | 76.91 | 4 | < 0.001 | 0.12 | 0.91 | 0.03 | 0.06 | 48.57 |
| EFA – between | 1194 | 157.75 | 4 | < 0.001 | 0.18 | 0.88 | 0.02 | 0.05 | 129.41 |
| Control Condition | 601 | 33.20 | 4 | < 0.001 | 0.11 | 0.94 | 0.02 | 0.04 | 7.61 |
| Mind-fulness Condition | 593 | 72.91 | 4 | < 0.001 | 0.17 | 0.84 | 0.03 | 0.06 | 47.37 |
| Two-level EFA (MPlus) | 1194 | 78.26 | 8 | < 0.001 | 0.09 | 0.92 | 0.04 (Within)^a^  0.03 (Between)^a^ | | 59918.63 |
| *Notes.* ^a^ MPlus outputs Standardized Root Mean Square Residual (SRMR); EFA = Exploratory Factor Analysis; RMSEA = Root Mean Square Error of Approximation; TLI = Tucker Lewis Index; RMSR = Root Mean Square of the Residuals; BIC = Bayesian Information Criterion | | | | | | | | | |

## Correlation Plots

| Figure S1  *Correlation Matrix of Primary and Secondary Variables* |
| --- |
| 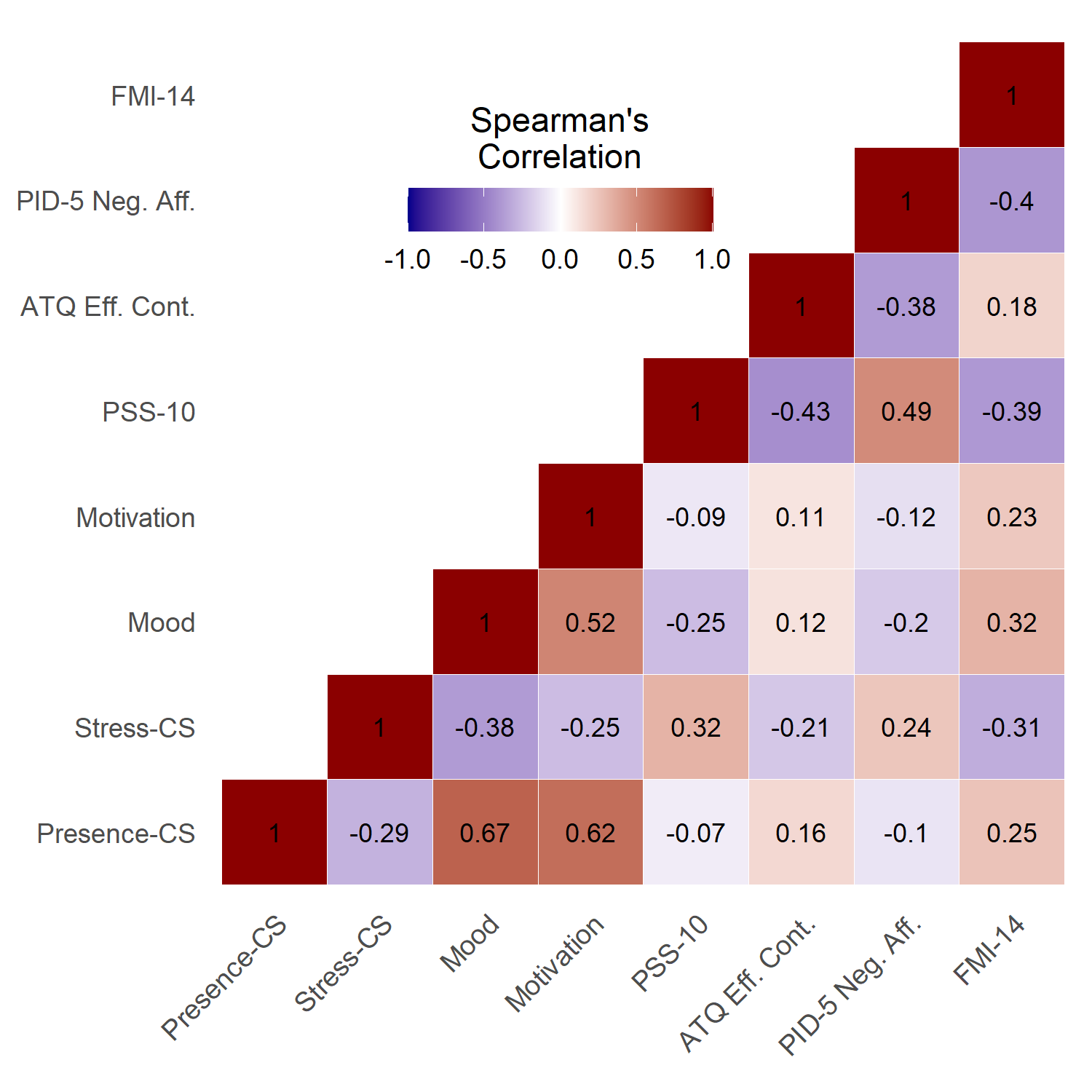 |
| *Note.* Spearman’s correlation coefficients are based on data of *n =* 244 students who reported baseline data of primary and secondary variables. FMI = Freiburg Mindfulness Inventory; ATQ Eff. Cont. = Adult Temperament Questionnaire – Effortful Control Subscale; PID-5 Neg. Aff. = Personality Inventory for DSM-5 – Negative Affectivity Subscale; PSS = Perceived Stress Scale; CS = Composite Score. |

| Figure S2  *Test-Retest Correlation Matrix* |
| --- |
| 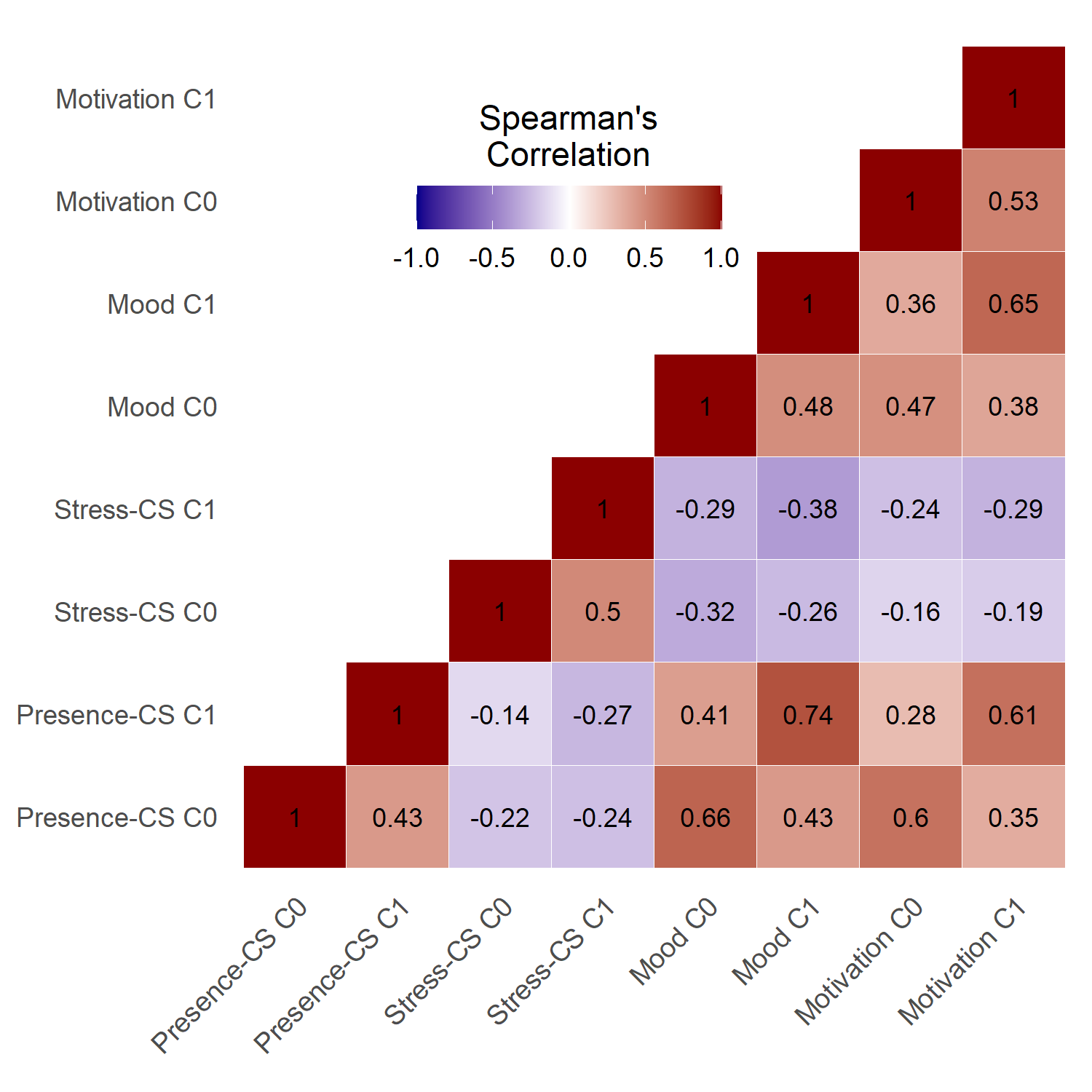 |
| *Note.* Spearman’s correlation coefficients are based on data of *n* = 170 students who reported two control observations; CS = Composite Score; C0 = Control condition at baseline (T0); C1 = Second control condition. |

# Additional descriptive statistics

| **Figure S3**  *Flow Chart of Lecturers’ Data Collection* |
| --- |
| 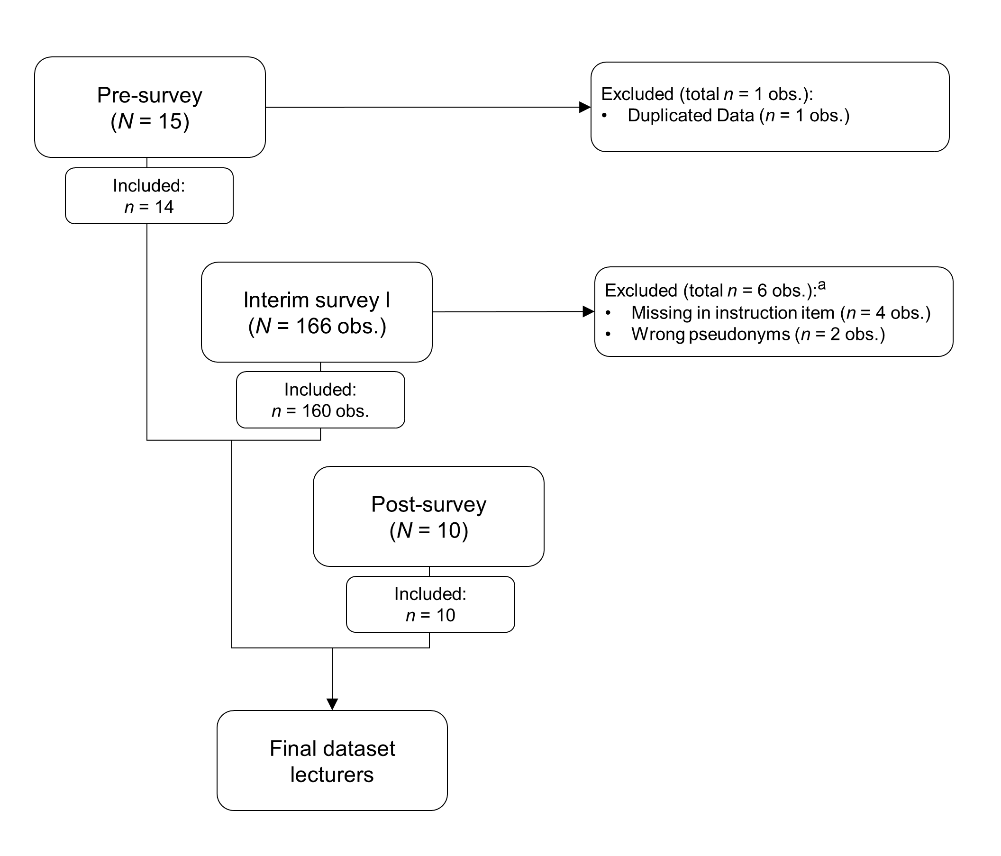 |
| *Note.* ^a^ We changed misspellings in n = 1 observation (pre-survey) and n = 6 observations (weekly survey) while merging pre-survey and weekly survey I. |

| Table S5  *Response Rates of Students Over the Course of the Semester* | | | | | | | | | | | | | |
| --- | --- | --- | --- | --- | --- | --- | --- | --- | --- | --- | --- | --- | --- |
|  | **Number of teaching sessions** | | | | | | | | | | | | **Total** |
|  | 1 | 2 | 3 | 4 | 5 | 6 | 7 | 8 | 9 | 10 | 11 | 12 |  |
| Control | 8 (0.5%) | 195 (13%) | 6 (0.4%) | 153 (10%) | 0 (0%) | 92 (6.3%) | 9 (0.6%) | 67 (4.6%) | 24 (1.6%) | 40 (2.7%) | 6 (0.4%) | 0 (0%) | 600 (41%) |
| Participation | 174 (12%) | 5 (0.3%) | 144 (9.8%) | 6 (0.4%) | 98 (6.7%) | 14 (1.0%) | 65 (4.4%) | 14 (1.0%) | 41 (2.8%) | 10 (0.7%) | 22 (1.5%) | 0 (0%) | 593 (41%) |
| No | 10 (0.7%) | 1 (<0.1%) | 15 (1.0%) | 0 (0%) | 7 (0.5%) | 2 (0.1%) | 4 (0.3%) | 7 (0.5%) | 3 (0.2%) | 3 (0.2%) | 9 (0.6%) | 0 (0%) | 61 (4.2%) |
| Partly | 82 (5.6%) | 2 (0.1%) | 39 (2.7%) | 1 (<0.1%) | 32 (2.2%) | 2 (0.1%) | 19 (1.3%) | 9 (0.6%) | 9 (0.6%) | 3 (0.2%) | 7 (0.5%) | 3 (0.2%) | 208 (14%) |
| Total | 274 (19%) | 203 (14%) | 204 (14%) | 160 (11%) | 137 (9.4%) | 110 (7.5%) | 97 (6.6%) | 97 (6.6%) | 77 (5.3%) | 56 (3.8%) | 44 (3.0%) | 3 (0.2%) | 1,462 (100%) |
| *Note.* Participation = full participation; No = no participation; partly = partly participation; Some of the courses did not have twelve teaching sessions. Response rates must be interpreted carefully. | | | | | | | | | | | | | |

# Additional exploratory analyses

| Table S6  *Standardized Regression Coefficients of Additional Exploratory Analyses* | | | | | | | | | | | | | | | | |
| --- | --- | --- | --- | --- | --- | --- | --- | --- | --- | --- | --- | --- | --- | --- | --- | --- |
|  | **Outcome variables** | | | | | | | | | | | | | | | |
|  | **Presence composite score** | | | | **Stress composite score** | | | | **Mood** | | | | **Motivation** | | | |
|  | *ß* | 95% CI | *p* | *R²/*  Cond. *R²* | *ß* | 95% CI | *p* | *R²/*  Cond. *R²* | *ß* | 95% CI | *p* | *R²/*  Cond. *R²* | *ß* | 95% CI | *p* | *R²/*  Cond.  *R²* |
| Model 1a-d: Normal sample incl. no and partial participation (*n* = 1462 obs.) |  |  |  | 0.07 / 0.44 |  |  |  | 0.04 / 0.46 |  |  |  | 0.04 / 0.51^a^ |  |  |  | 0.05 / 0.58^a^ |
| (Intercept) | -0.22 | -0.34, -0.10 | **<0.001** |  | 0.12 | -0.02, 0.26 | 0.089 |  | -0.12 | -0.22, -0.02 | **0.020** |  | -0.09 | -0.27, 0.08 | 0.297 |  |
| condition [full vs. control] | 0.55 | 0.46, 0.64 | **<0.001** |  | -0.38 | -0.47, -0.29 | **<0.001** |  | 0.39 | 0.30, 0.47 | **<0.001** |  | 0.43 | 0.35, 0.51 | **<0.001** |  |
| condition [no vs. control] | -0.13 | -0.35, 0.10 | 0.272 |  | 0.33 | 0.10, 0.55 | **0.004** |  | -0.23 | -0.45, -0.02 | **0.036** |  | -0.22 | -0.42, -0.02 | **0.030** |  |
| condition [partly vs. control] | 0.07 | -0.06, 0.20 | 0.305 |  | -0.04 | -0.17, 0.09 | 0.523 |  | 0.06 | -0.07, 0.18 | 0.382 |  | 0.10 | -0.02, 0.22 | 0.094 |  |
| Model 2a-d: Mindfulness subsample (*n* = 829 obs.) |  |  |  | 0.15 / 0.50 |  |  |  | 0.09 / 0.50 |  |  |  | 0.13 / 0.60^a^ |  |  |  | 0.20 / 0.51 |
| (Intercept) | 0.19 | 0.09, 0.30 | **<0.001** |  | -0.15 | -0.30, -0.01 | **0.042** |  | 0.16 | 0.05, 0.26 | **0.005** |  | 0.14 | 0.01, 0.26 | **0.039** |  |
| condition [no vs. full] | -0.38 | -0.90, 0.13 | 0.147 |  | 1.15 | 0.59, 1.71 | **<0.001** |  | -0.66 | -1.18, -0.14 | **0.012** |  | -0.20 | -0.72, 0.32 | 0.456 |  |
| condition [partly vs. full] | -0.30 | -0.45, -0.16 | **<0.001** |  | 0.19 | 0.04, 0.35 | **0.017** |  | -0.11 | -0.26, 0.04 | 0.138 |  | -0.05 | -0.20, 0.10 | 0.494 |  |
| exercise motivation | 0.78 | 0.60, 0.96 | **<0.001** |  | -0.60 | -0.80, -0.40 | **<0.001** |  | 0.78 | 0.59, 0.97 | **<0.001** |  | 0.93 | 0.75, 1.12 | **<0.001** |  |
| condition [no vs. full] x  exercise motivation | -0.42 | -0.93, 0.09 | 0.104 |  | 1.07 | 0.52, 1.63 | **<0.001** |  | -0.72 | -1.24, -0.21 | **0.006** |  | -0.35 | -0.87, 0.16 | 0.178 |  |
| condition [partly vs. full] x exercise motivation | -0.59 | -0.89, -0.29 | **<0.001** |  | 0.28 | -0.04, 0.61 | 0.085 |  | -0.23 | -0.54, 0.07 | 0.133 |  | -0.15 | -0.45, 0.15 | 0.336 |  |

| Table S6 (continued)  *Standardized Regression Coefficients of Additional Exploratory Analyses* | | | | | | | | | | | | | | | | |
| --- | --- | --- | --- | --- | --- | --- | --- | --- | --- | --- | --- | --- | --- | --- | --- | --- |
|  | **Outcome variables** | | | | | | | | | | | | | | | |
|  | **Presence composite score** | | | | **Stress composite score** | | | | **Mood** | | | | **Motivation** | | | |
|  | *ß* | 95% CI | *p* | *R²/*  Cond. *R²* | *ß* | 95% CI | *p* | *R²/*  Cond. *R²* | *ß* | 95% CI | *p* | *R²/*  Cond. *R²* | *ß* | 95% CI | *p* | *R²/*  Cond.  *R²* |
| Model 3a-d: Online subsample (*n* = 929) |  |  |  | .09/ .51 |  |  |  | .05/ .47 |  |  |  | .05/ .48 |  |  |  | .06/  .56 |
| (Intercept) | -0.65 | -1.15, -0.15 | **0.010** |  | 0.83 | 0.32, 1.34 | **0.002** |  | -0.79 | -1.30, -0.27 | **0.003** |  | -0.61 | -1.12, -0.10 | **0.019** |  |
| condition [full vs. control] | 0.59 | 0.49, 0.69 | **<0.001** |  | -0.38 | -0.48, -0.27 | **<0.001** |  | 0.42 | 0.32, 0.53 | **<0.001** |  | 0.47 | 0.37, 0.57 | **<0.001** |  |
| camera [camera on vs off] | 0.17 | -0.11, 0.46 | 0.240 |  | 0.30 | 0.01, 0.60 | **0.042** |  | 0.12 | -0.18, 0.41 | 0.429 |  | 0.39 | 0.10, 0.68 | **0.008** |  |
| internet [ok instable vs disturbing] | 0.29 | -0.29, 0.86 | 0.327 |  | -0.79 | -1.38, -0.20 | **0.009** |  | 0.49 | -0.10, 1.08 | 0.101 |  | 0.55 | -0.01, 1.12 | 0.055 |  |
| internet [stable vs. disturbing] | 0.41 | -0.07, 0.90 | 0.095 |  | -0.74 | -1.24, -0.24 | **0.004** |  | 0.63 | 0.14, 1.13 | **0.013** |  | 0.41 | -0.07, 0.89 | 0.092 |  |
| Model 4a-d: Normal sample  (*n* = 1193) |  |  |  | .08/ .45 |  |  |  | .05/ .49^a^ |  |  |  | .04/ .49 |  |  |  | .06/  .54 |
| (Intercept) | -0.22 | -0.38, -0.05 | **0.010** |  | 0.07 | -0.11, 0.25 | 0.428 |  | -0.13 | -0.30, 0.04 | 0.142 |  | -0.03 | -0.24, 0.18 | 0.767 |  |
| condition [full vs. control] | 0.51 | 0.34, 0.69 | **<0.001** |  | -0.56 | -0.74, -0.38 | **<0.001** |  | 0.53 | 0.35, 0.70 | **<0.001** |  | 0.53 | 0.36, 0.70 | **<0.001** |  |
| time per condition | -0.01 | -0.06, 0.05 | 0.795 |  | 0.05 | -0.01, 0.11 | 0.093 |  | -0.02 | -0.08, 0.03 | 0.423 |  | -0.07 | -0.12, -0.01 | **0.014** |  |
| condition [full vs. control] x time per condition | 0.02 | -0.05, 0.09 | 0.592 |  | 0.09 | 0.01, 0.16 | **0.021** |  | -0.06 | -0.13, 0.01 | 0.100 |  | -0.04 | -0.11, 0.03 | 0.211 |  |
| Model 5a-d: Normal sample  (*n* = 1153) |  |  |  | .10/ .46 |  |  |  | .04/ .44 |  |  |  | .05/ .52^a^ |  |  |  | .06/  .54 |
| (Intercept) | -0.24 | -0.36, -0.12 | **<0.001** |  | 0.15 | 0.00, 0.30 | **0.047** |  | -0.13 | -0.24, -0.02 | **0.016** |  | -0.15 | -0.34, 0.05 | 0.135 |  |
| condition [full vs. control] | 0.56 | 0.47, 0.65 | **<0.001** |  | -0.38 | -0.47, -0.29 | **<0.001** |  | 0.39 | 0.31, 0.48 | **<0.001** |  | 0.45 | 0.37, 0.54 | **<0.001** |  |
| lecturers’ instruction experience | -0.03 | -0.31, 0.24 | 0.808 |  | 0.14 | -0.21, 0.49 | 0.440 |  | 0.08 | -0.15, 0.31 | 0.507 |  | -0.19 | -0.66, 0.28 | 0.435 |  |
| lecturers‘ meditation experience | 0.23 | -0.07, 0.53 | 0.130 |  | -0.07 | -0.45, 0.31 | 0.710 |  | 0.16 | -0.10, 0.41 | 0.227 |  | 0.26 | -0.26, 0.78 | 0.335 |  |
| lecturers‘ meditation frequency | -0.09 | -0.32, 0.13 | 0.408 |  | -0.15 | -0.42, 0.13 | 0.297 |  | 0.10 | -0.10, 0.30 | 0.342 |  | -0.08 | -0.44, 0.28 | 0.670 |  |
| *Note.* ^a^ Robust model is displayed; Normal sample refers to student observations of control and full participation. Note that in models 6a-d there were *n =* 40 missing observations; Mindfulness subsample refers to the sample without the control condition; Online subsample only includes online lectures. | | | | | | | | | | | | | | | | |

# References

1. Lüdecke, D., Ben-Shachar, M. S., Patil, I. & Makowski, D. Extracting, computing and exploring the parameters of statistical models using R. *Journal of Open Source Software* **5**, 2445 (2020).

2. Kim, E. S., Dedrick, R. F., Cao, C. & Ferron, J. M. Multilevel factor analysis: Reporting guidelines and a review of reporting practices. *Multivariate Behavioral Research* 0–0 (2016) doi:10.1080/00273171.2016.1228042.

3. Baayen, R. H., Davidson, D. J. & Bates, D. M. Mixed-effects modeling with crossed random effects for subjects and items. *Journal of Memory and Language* **59**, 390–412 (2008).

4. Muthén, B. Latent variable modeling of longitudinal and multilevel data. *Sociological Methodology* **27**, 453–480 (1997).

5. Makowski, D., Lüdecke, D., Patil, I., Ben-Shachar, M. S. & Wiernik, B. M. datawizard: Easy data wrangling. *CRAN* (2021).
